# Evidence of Unprecedented High Electronic Conductivity in Mammalian Pigment Based Eumelanin Thin Films After Thermal Annealing in Vacuum

**DOI:** 10.3389/fchem.2019.00162

**Published:** 2019-03-26

**Authors:** Ludovico Migliaccio, Paola Manini, Davide Altamura, Cinzia Giannini, Paolo Tassini, Maria Grazia Maglione, Carla Minarini, Alessandro Pezzella

**Affiliations:** ^1^Department of Chemical Sciences, University of Naples “Federico II”, Naples, Italy; ^2^Istituto di Cristallografia (IC), CNR, Bari, Italy; ^3^Laboratory for Nanomaterials and Devices (SSPT-PROMAS-NANO), ENEA C. R. Portici, Piazzale Enrico Fermi 1, Località Granatello, Portici, Italy; ^4^Institute for Polymers, Composites and Biomaterials (IPCB), CNR, Pozzuoli, Italy

**Keywords:** charge transport, vacuum thermal treatment, electrical conductivity, organic (bio)electronics, eumelanin, melanins, molecular orientation

## Abstract

Melanin denotes a variety of mammalian pigments, including the dark electrically conductive eumelanin and the reddish, sulfur-containing, pheomelanin. Organic (bio)electronics is showing increasing interests in eumelanin exploitation, e.g., for bio-interfaces, but the low conductivity of the material is limiting the development of eumelanin-based devices. Here, for the first time, we report an abrupt increase of the eumelanin electrical conductivity, revealing the highest value presented to date of 318 S/cm. This result, obtained via simple thermal annealing in vacuum of the material, designed on the base of the knowledge of the eumelanin chemical properties, also discloses the actual electronic nature of this material's conduction.

## Introduction

In the 1974, McGinness et al. reported the first experimental evidence of the semiconducting behavior of the eumelanin (McGinness et al., 1974), the polyindolic pigment responsible, inter alia, of the dark-brown pigmentation of the mammalian (including human) skin, hair, and iris. The study followed a pioneering suggestion by Pullman and Pullman (1961) on the possible existence of energy bands associated with a non-localized empty molecular orbital within an infinite eumelanin polymer chain acting as an one-dimensional semiconductor.

Since then, the charge transport properties of this challenging materials class were extensively studied (d'Ischia et al., 2015), and particularly in the recent years, after the renewed interest in the topic, because of the prospect to exploit the eumelanin in organic (bio)electronics (Muskovich and Bettinger, 2012). Since the ′70 also the chemical characterization of eumelanin pigment witnessed a noteworthy development allowing to delineate a sound picture of the chemical structural signatures of the natural pigment and to design and fabricate valuable synthetic models (d'Ischia et al., 2015). Among these models the one involving oxidative polymerization of the 5,6-dihydroxyindole (DHI, Figure S1), the ultimate monomer precursor in the formation pathways of natural eumelanin, proved to be one of the most valuable for both its chemical structure and biocompatibility and is widely acknowledged (d'Ischia et al., 2015). To date, conductivity of synthetic as well as natural eumelanin is reported in the range (Osak et al., 1989; Meredith and Sarna, 2006) 10^−13^−10^−5^ S/cm, largely depending on the measuring conditions, and especially on the presence of humidity in the measuring environment (Jastrzebska et al., 1995). For valuable applications, higher conductivity values are needed yet, thus several studies explored the integration of the eumelanin with other more conductive materials (Mihai et al., 2013; Gargiulo et al., 2015; Migliaccio et al., 2017), but strongly affecting its chemistry, or exploiting severe modifications of eumelanin-like materials to gain a graphene-like material, as for example by pyrolytic treatment of polydopamine under hydrogen or argon atmosphere (Kong et al., 2012; Li et al., 2013). Although the mechanisms of the charge transport in the eumelanin are still not fully clear, several evidences are concurring to sustain a hybrid ionic-electronic behavior (Mostert et al., 2012; Wünsche et al., 2015), where the electronic contribution depends on the presence, extent and the redox properties (Mostert et al., 2012) of the delocalized aromatic systems, while the ionic part is largely dictated by the hydration level of the material (Wünsche et al., 2015) (i.e., the humidity in the measuring environment).

Basing on the concurring evidences disclosing the correlation between the chemical-physical properties of the eumelanin and the polyindole π-system stacking, as well as the packing of molecular constituents within the material (Pezzella et al., 2009; Bonavolontà et al., 2017), we speculated about the modulation of the electronic conductivity (Noriega et al., 2013; Liu et al., 2017) by acting on the polyindole packing in eumelanin thin films. This is bringing us, here, for the first time in our knowledge, to report the preparation and characterization of eumelanin thin films showing the highest conductivity values of this material up to 318 S/cm.

Conductive eumelanin films were prepared via the preliminary oxidative polymerization of the solid state form of DHI (d'Ischia et al., 2013) and then by thermal annealing of the material films, at temperatures not higher than 600°C and under high vacuum conditions (order of 10^−6^ mbar). We name the obtained material as High Vacuum Annealed Eumelanin, HVAE.

## Experimental Part

All the commercially available reagents and materials were used as received. All the solvents were analytical grade quality. The DHI was prepared according to a reported procedure (d'Ischia et al., 2013). The samples were prepared on quartz substrates (dimensions 15 mm × 6 mm × 1.2 mm), cleaned by sonication in a solution of detergent Borer Chemie AG Deconex 12PA® in deionized water (18 MΩ·cm) at 70°C for 30 min, and rinsed in deionized water, then in acetone and then in isopropanol for 15 min each sequentially. A concentrated solution of DHI in methanol-ethyl acetate (1:1 v/v) (50 mg/mL) was prepared, filtered through a 0.2 μm Whatman membrane before deposition; on each sample, 15 μL of this solution were applied. Thin films were obtained by spin coating, using a Laurell WS-650MZ23NPP/LITE coater, with the spinning recipe: acceleration 2,000 rpm/s, speed 3,500 rpm, duration 30 s. The resulting films were dried at 90°C for 30 min in oven in air. The thicknesses of the DHI films were 230 ± 10 nm, measured using a stylus profilometer KLA Tencor P-10. Thermogravimetric analysis (TGA) performed under not oxidizing atmosphere using a Perkin–Elmer Pyris thermogravimetric analyzer. Scanning electronic microscopy (SEM) was run using a SEM Zeiss Leo 1530 Gemini. UV-Vis spectra were recorded using a Perkin-Elmer Lambda 900 spectrophotometer. The electron paramagnetic resonance (EPR) spectra were measured using an X-band (9 GHz) Bruker Elexys E-500 spectrometer, equipped with a superhigh sensitivity probe head), the Raman spectra were reordered Renishaw inVia Raman microscope (532 nm), which uses a microscope to focus a laser source onto specific areas of a sample, then the light scattered off the surface of the sample is collected and directed to a Raman spectrometer), the FTIR analysis were run using a Thermo Fischer Scientific Nicolet 6700 FTIR to determine the attenuated total reflectance (ATR) spectra of the samples, with a resolution of 4 cm^−1^ and 16 scans averaged for each spectrum in a range between 4000 and 650 cm^−1^), MALDI-MS analysis was done using a positive reflectron MALDI and LDI spectra were recorded on a Sciex 4800 MALDI ToF/ToF instrument. Grazing Incidence Wide Angle X-ray Scattering (GIWAXS) was run with a Fr-E+ SuperBright rotating anode microsource (CuKa, λ = 0.154 nm) equipped to a three-pinhole camera (Rigaku SMAX-3000) through a multilayer focusing optics (Confocal Max-Flux; CMF 15–105). Elemental composition (C, H, N wt.%) was estimated using a Perkin–Elmer 2400 CHNSO elemental analyzer. Measurements of electrical resistance vs. temperature were performed measuring the two-terminals devices of one type of the HVAE (600°C, 2 h, 10^−6^ mbar) in a probe station CASCADE Summit 11000B-M, featuring a closed chamber with thermal chuck, keeping the samples in constant flow (10 L/min) of pure dry Nitrogen, allowing the temperature to stabilize within ±1°C before each measurements run, and using a Keithley 4200 SCS Semiconductor Characterization System to acquire the electrical data.

The eumelanin formation was obtained by the oxidation of the DHI films thanks to the Ammonia-Induced Solid State Polymerization (AISSP) method, a recently developed solid state protocol (d'Ischia et al., 2013; Pezzella et al., 2015). Each sample was exposed for 12 h to an oxidizing atmosphere made of oxygen, water and ammonia vapors at controlled temperature (25°C), produced by the equilibrium of the air with an ammonia solution (5% NH_3_ in H_2_O) in a sealed chamber at 1 bar pressure. The material so obtained is here named DHI-eumelanin, to distinguish it from the starting DHI, and from the final HVAE. The thicknesses of the DHI-eumelanin films were 260 ± 6 nm. Films showed the typical dark brown color of the eumelanin (Figure S2), presenting flat surfaces (Figure S3, Table S1; surfaces roughness images were taken using a Taylor Hobson® CCI-HD non-contact 3D Optical Profilometer with thin & thick film measurement capability; films' roughness was estimated as a Root Mean Square (RMS) value from several scans on each sample).

The DHI-eumelanin films were finally turned into HVAE by annealing at different controlled temperatures (230, 300, 450, and 600°C, ±1°C for each value) in high vacuum conditions (10^−6^ mbar); some samples were also annealed at various time lengths (from 30 min up to 6 h). The processes were performed in a dedicated high vacuum chamber using a turbomolecular pump to obtain the vacuum level, and doing preliminary leak detection and samples temperature verifications. The mean thickness of the HVAE films was dependent on the annealing conditions, with the smallest values down to 110 ± 2 nm for the processes at 600°C longer than 1 h (Figure S5).

## Results and Discussion

The chosen annealing temperatures were well below the values reported as the starting temperature for the degradation (Albano et al., 2016) and/or the carbonization processes in similar materials (Yu et al., 2014), but includes a significant part of the eumelanin mass loss region, as shown by thermogravimetric analysis (TGA). Moreover, applied temperatures include the complete loss of both weakly and strongly bound water (Albanese et al., 1984; Meredith and Sarna, 2006; Albano et al., 2016), as well as the loss of CO_2_ from carboxyl groups in DHI-eumelanin (thermal decarboxylation) (Swan and Waggott, 1970). Indeed, TGA data under not oxidizing conditions indicate that mass loss is nearly completed at 800°C, suggesting that little or no modification of the molecular backbone occurs at 600°C. Instead, a complete different picture is obtained in presence of oxygen, which critically affects the stability of the material (Figure S4).

Morphology and surface analysis of the materials at the different stages of the process revealed a nearly unmodified roughness, passing from the starting DHI films to the HVAE films (Figure S3) (using the definition of the roughness according to the standard ISO 25178; DHI roughness = 6.45 nm; DHI-eumelanin roughness = 6.52 nm; HVAE roughness = 6.58 nm), while, as said, the thickness suffered a significant decrease in function of the annealing temperature from 260 to 109 nm in the case of the sample treated at 600°C (Figure S5). This was expected because of the said tendency of the eumelanin to loss labile carboxylic groups (Swan and Waggott, 1970; d'Ischia et al., 2013; Albano et al., 2016) and on the possible loss of low molecular weight components embedded in the material.

Scanning electronic microscopy (SEM) inspection confirmed the retaining of the high quality morphology of the HVAE films (Figure S6), showing an uniform surface of this material.

**Figure 1:**
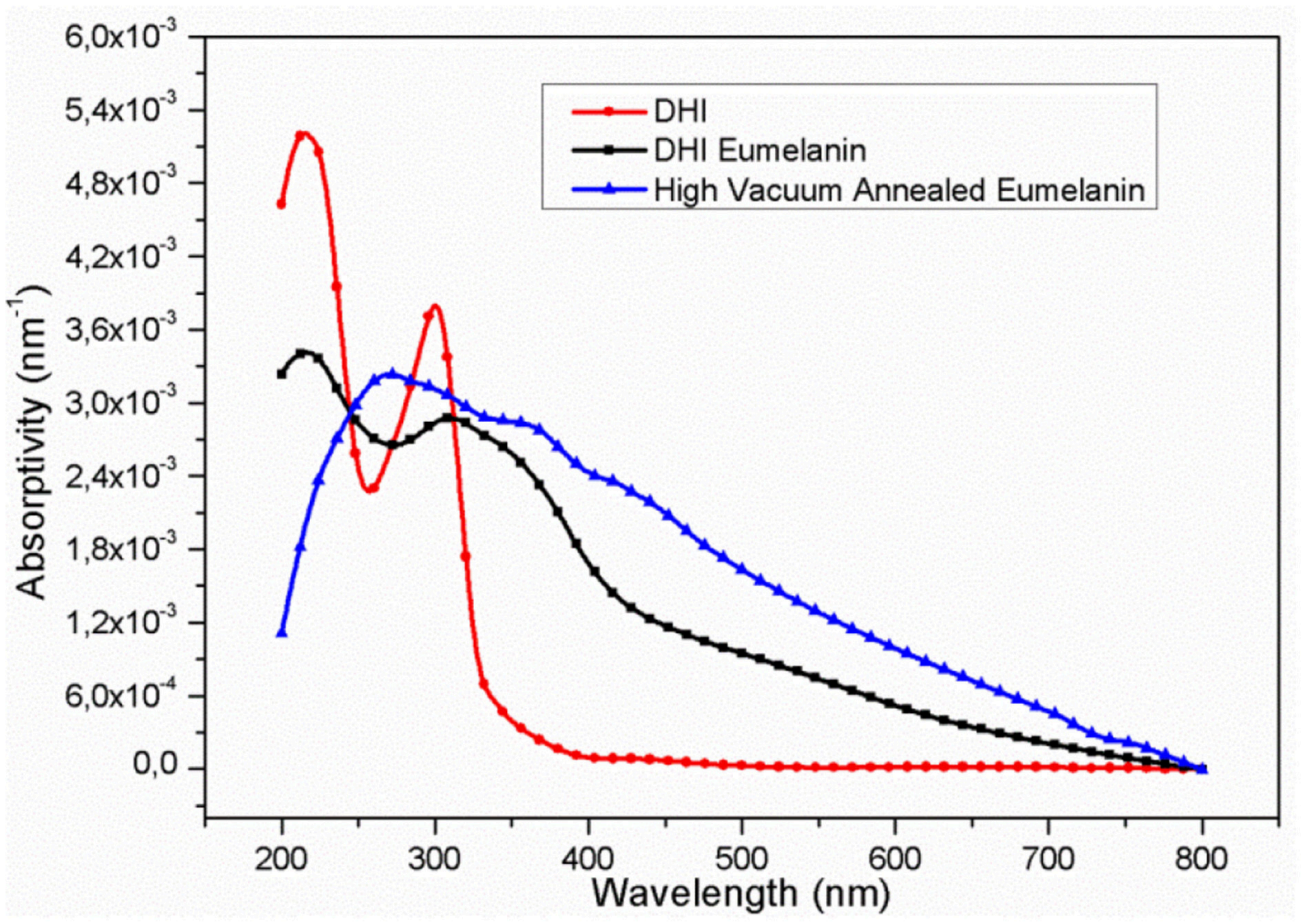
UV-Vis absorptivity (percent absorbance/film thickness) of the films at the different process stages: (red, circles) DHI; (black, squares) DHI-eumelanin (film after AISSP); (blue, triangles) HVAE (film after thermal annealing in vacuum: 600°C; 2 h; 10^−6^ mbar).

UV-Vis spectra, observed at the different process steps (Figure 1), show an evident increase in the absorption coefficients in nearly the entire UV-Vis range, passing from the DHI to the DHI-eumelanin and to the HVAE. This phenomenon is associated to the increase of both the delocalization of the aromatic systems and their π-stacking interactions (Pezzella et al., 2009; Bonavolontà et al., 2017), that suggest the actual increase of the extension and of the filling factor (Albanese et al., 1984; Bonavolontà et al., 2017) for the delocalized aromatic systems of the material backbone, in particular happening after the thermal annealing in vacuum: i.e., this reorganization results in an overlap of the π-electronic density of the adjacent packed chains and the delocalization of their electronic wave-functions (Koller et al., 2007).

**Figure 2:**
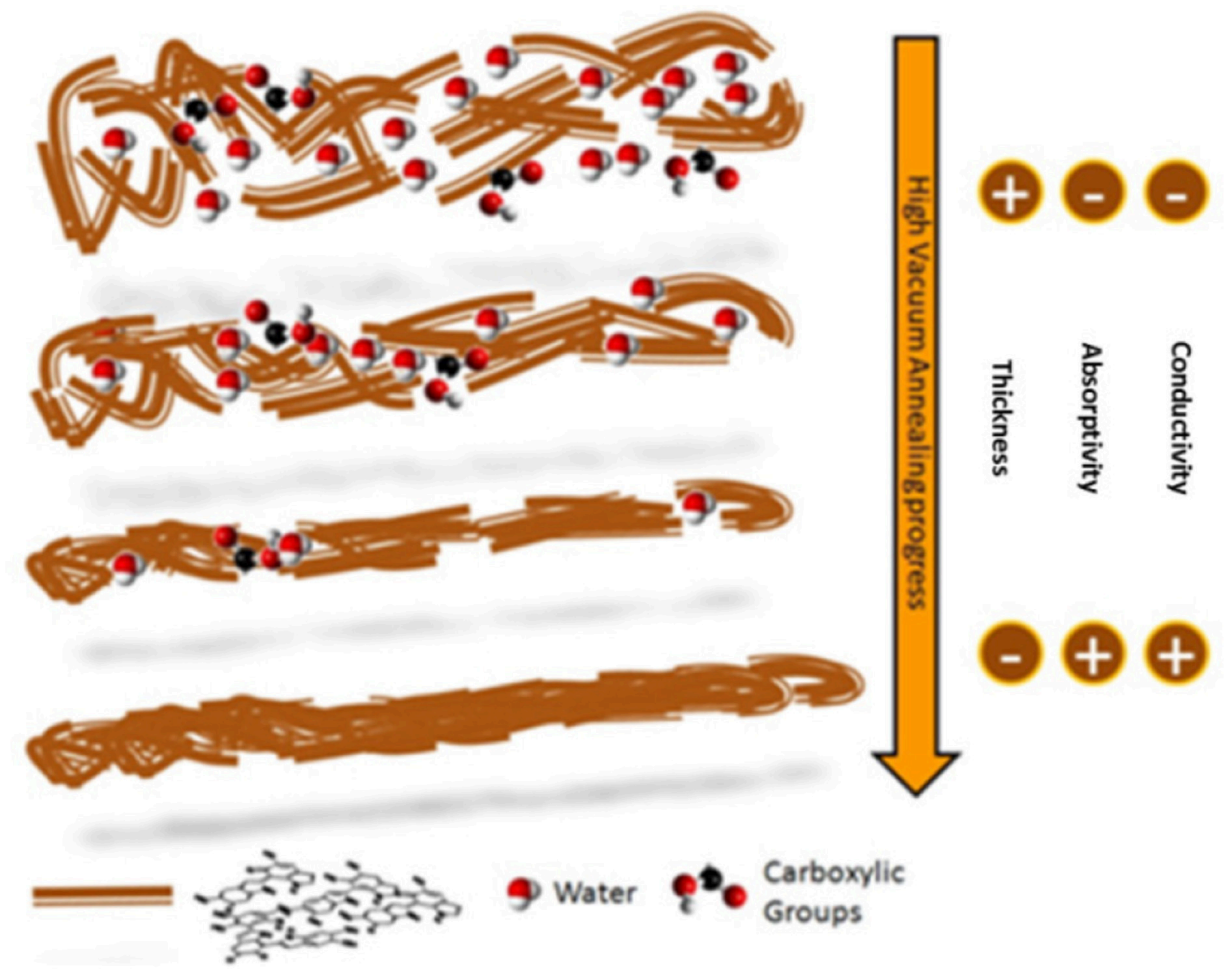
Pictorial model of the polyindole packing evolution during the high vacuum annealing. Water molecules and carboxylic groups are evidenced, to show their reduction in the material as the process temperature increases.

Strong support to the picture of a structural reorganization and an enhanced packing order (Roncali and Thobie-Gautier, 1994; Roncali, 1997; Liu et al., 2016) of the molecular constituents within the HVAE films was further given by the retaining of the typical eumelanin signature observed using different characterization techniques (Figures S7–S10): (i) the electron paramagnetic resonance (EPR) spectrum (Meredith and Sarna, 2006; d'Ischia et al., 2013), (ii) the Raman spectroscopy (Capozzi et al., 2005; Albano et al., 2016), (iii) the FTIR analysis (Hyogo et al., 2011), and (iv) the MALDI-MS (Pezzella et al., 2015) analysis. A pictorial representation of this packing model, made possible by the concomitant loss of labile and low molecular weight components (Swan and Waggott, 1970) and by the clustering of the longer polyindole chains, is shown in Figure 2.

Although each of these techniques cannot be considered conclusive to confirm the nature of the molecular constituents within the films, the concurrence of data from different spectroscopies has to be considered decisive, basing on literature. Indeed, coherently with UV-Vis profiles, also ERP spectra of HVAE (Figure S7) were consistent with those reported in literature for eumelanin samples featuring a single, roughly symmetric signal at a *B* value in the range of 2.003 ± 0.004 G, a typical signature of the eumelanin pigment (Meredith and Sarna, 2006), associated to carbon-centered radicals formed in the 5,6-dihydroxyindole polymerization.

Without entering into the details of the Raman spectra (Figure S8), it is worth to note here how the comparison of the profiles before and after the annealing reveals, in agreement with the loss of carboxylic groups and possible pyrrolic acids, a relative reduction of the G band (the range of 1,600 cm^−1^) following the reduction of O and N contribution. Consistent information is provided by the FTIR spectra of the DHI-eumelanin and the HVAE films (Figure S9) too, highlighting in particular the drastic decrease of the signals associated to the C = O stretching (1,620 cm^−1^) and to the water (3,200 cm^−1^) (Hyogo et al., 2011).

Consistently with literature, MALDI profiles (Figure S10) of DHI-eumelanin and HVAE also share the recurring profile of masses of general formula *DHI oligomer*+*mO*_2_*-nCO*_2_ (Pezzella et al., 2015).

**Figure 3:**
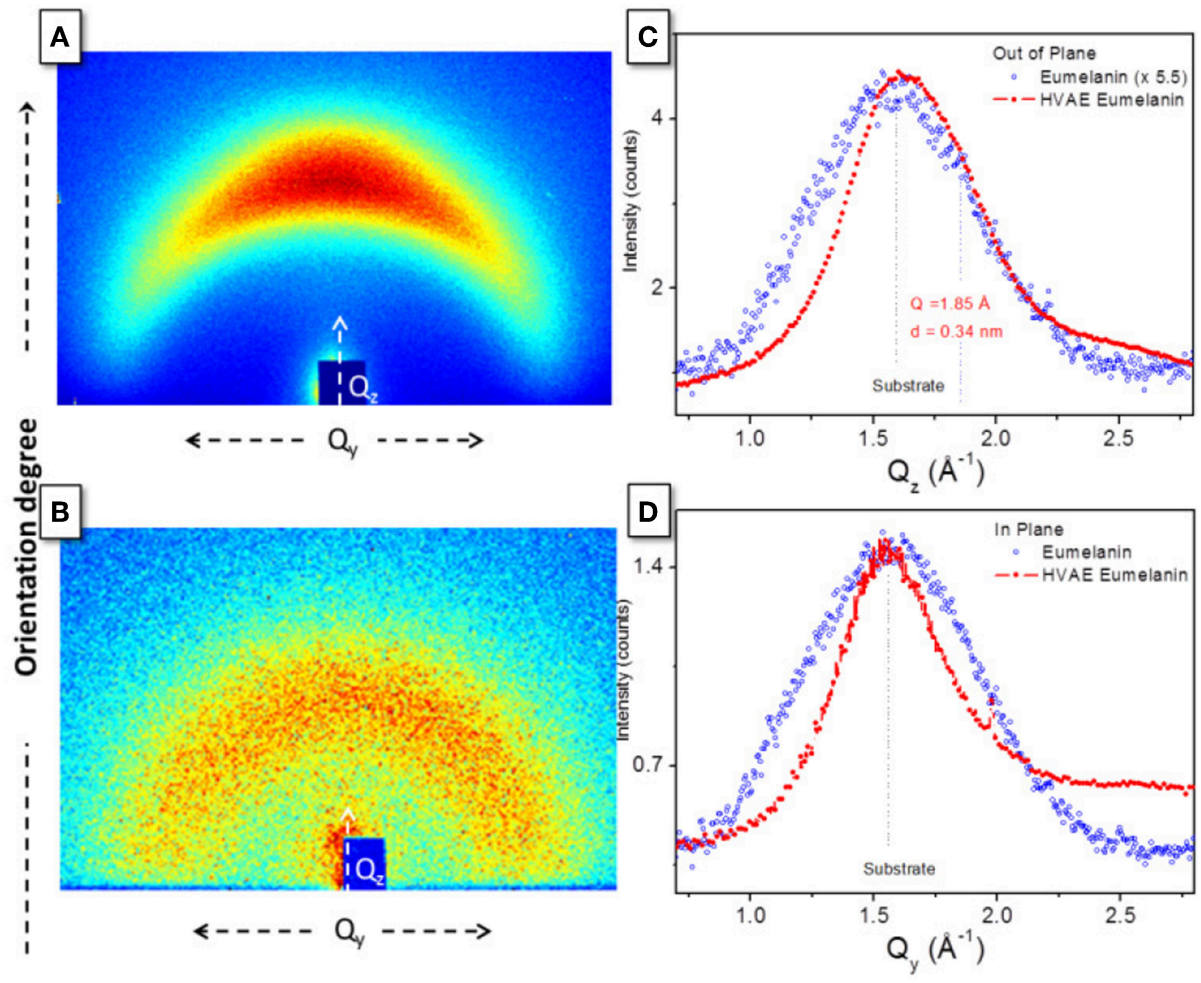
GIWAXS 2D patterns of **(A)** HVAE film (processed at 600°C for 2 h) and **(B)** DHI-eumelanin film. 1D radial cuts along **(C)** the out-of-plane and **(D)** the in-plane directions, obtained from the 2D maps in **(A,B)**.

Finally, a direct support to the packing evolution hypothesis comes from 2D GIWAXS patterns (Figure 3). Diffraction data were collected from as-prepared films deposited on glass substrates in Grazing Incidence Wide Angle X-ray Scattering. The different anisotropy degree of the intensity distribution along the diffraction rings indicates an increased orientation degree after the vacuum thermal treatment is operated. In particular, the HVAE film (Figure 3A) features a diffraction intensity definitely concentrated along the Q_z_ axis, i.e., perpendicularly to the sample surface, denoting a preferred orientation of the diffracting planes parallel to the film surface. On the other hand, the DHI-eumelanin film (Figure 3B) features a weak diffraction intensity evenly distributed along the azimuth of a broad diffraction ring, indicating low crystallinity and random orientation of the molecules. The 1D radial cuts extracted from the GIWAXS maps along the out-of-plane (Figure 3C) and in-plane (Figure 3D) directions show indeed a clear difference between the two directions in the case of the HVAE film: a peak asymmetry in the out-of-plane direction reveals a diffraction contribution of the oriented molecules appearing as a shoulder at q = 1.85 Å, at the side of the main peak at q = 1.56 Å which is ascribed to the substrate and is in turn the only scattering contribution in the in-plane cut. The shoulder in the out-of-plane direction is a clear signature of the formation of a well oriented stack, compatible with the expected supramolecular structure with 3.4 Å periodicity (Zajac et al., 1994; Chen et al., 2013).

On the contrary, in the case of the DHI-eumelanin film no difference between the diffraction intensities in the two directions is recognized (so that a 5.5 additional scale factor has been applied in Figure 3C for the sake of clarity in the comparison).

The electrical properties of the materials were measured using two set-ups, because of the different conductivity values that samples presented. Between the various measurements runs, the samples were stored in mild vacuum (10^−4^ mbar), cleaning the storage chamber each time it was opened using pure dry nitrogen (oxygen and water vapor content below 5 ppm).

A four-point probes system (Schroder, 1986; Bothma et al., 2008) Napson RESISTAGE RG-80 was used, measuring the sheet resistance of each film, and assuming that input current flowed through the full thickness of each thin layer, in order to calculate the material conductivity thanks to the films thickness. In this measurement configuration, the contact resistance between the probes and the material can be disregarded, because (i) the input current is fixed and known by the instrument, and it could flow in the device regardless the presence of a contact resistance in this circuit branch, allowing to obtain the sheet resistance value, and (ii) virtually no current passes in the voltmeter circuit branch, so a contact resistance (if it were present) in this point gives no effect in the measurements.

For low conductivity samples, the four-point probes Napson instrument cannot be used, so two-terminal devices were prepared with the different films of the materials, using silver paste to prepare the devices contact pads and cutting away the film where it was not necessary, to avoid anomalous current paths. A power supply source meter Keithley 2410 was used in four-point probes configuration, and the devices resistance was derived through the alternating polarity method (Daire, 2001), and then the conductivity calculated.

**Figure 4:**
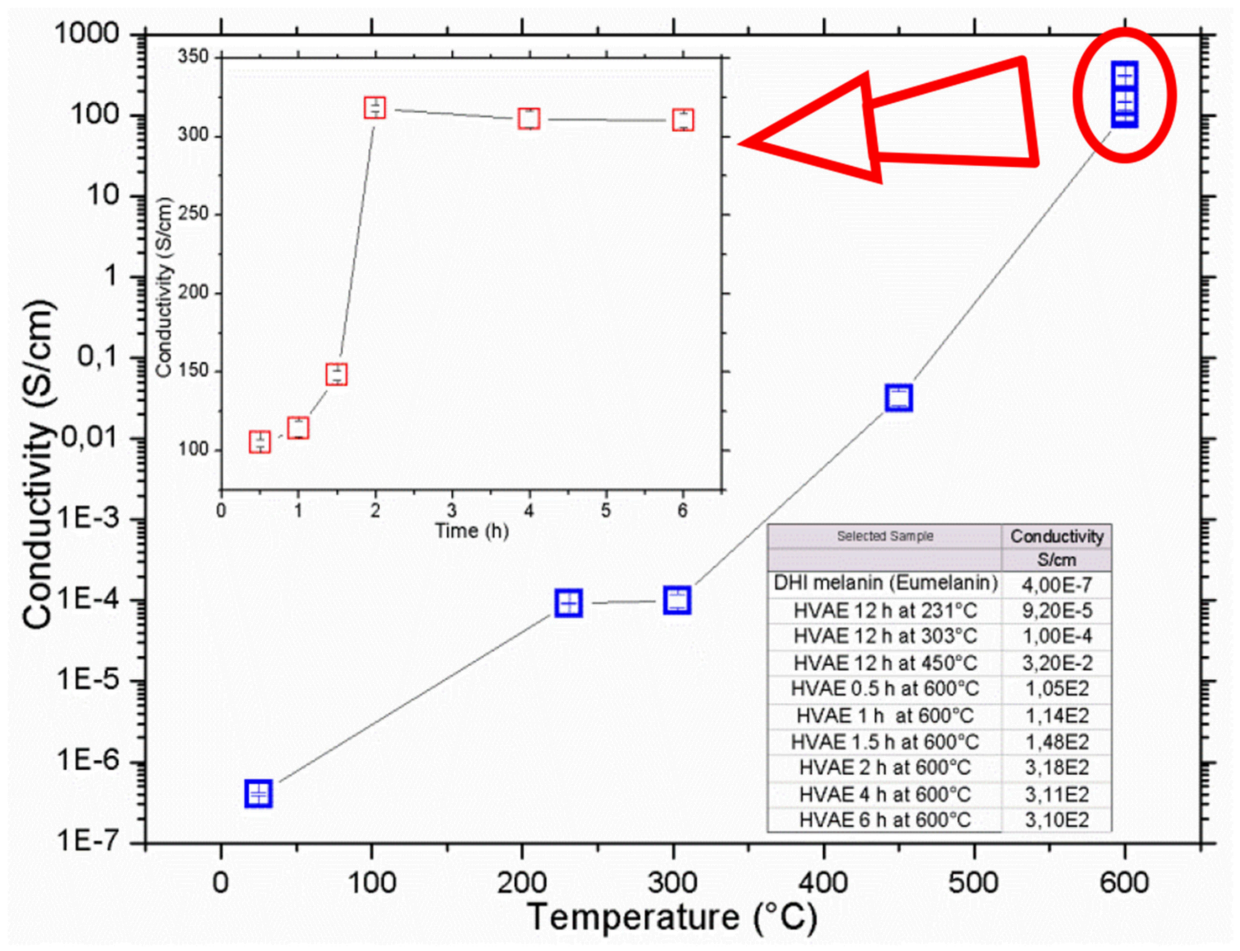
Conductivity of vacuum annealed eumelanin thin films vs. the annealing temperature and (inset) vs. the annealing time at 600°C temperature. Data are listed in the table. All the measurements were performed in air at room temperature. Errors of each point are indicated inside the plots symbols.

In Figure 4, the samples conductivity vs. the annealing temperature and vs. the duration of the processes is shown. After the vacuum annealing, the conductivity of the films featured a remarkable increase, up to over 9 orders of magnitude, passing from around 10^−7^ S/cm for the DHI and DHI-eumelanin films, up to an unprecedented value of 318 S/cm for the material processed at 600°C for 2 h, and anyway obtaining values larger than 100 S/cm for all the samples processed at 600°C (Figure 4 inset).

This unprecedented result is not a humidity response effect, as the data acquisitions were performed in few tens of seconds for each sample, with no variation of the ambient relative humidity, so suggesting that actual nature of the involved charge carriers is electronic. Nonetheless, at fixed temperature in air for longer time, the conductivity of the HVAE films appears pretty stable with time (Figure S13), allowing the material to sustain a constant current with a very low increase in the applied voltage along the time, as it can be expected for electronic conductive organics (Le et al., 2017). Even more, if the environmental humidity was being absorbed by the material films during the measurements, according to the current literature about eumelanin, its conductivity should be increased, which means that voltage should decrease during this type of measurement. Instead, here it can be observed the opposite effect: i.e., the sample resistance was increasing along the time. So, HVAE conduction can be considered largely independent from the presence of humidity in the material.

Current-voltage measurements performed before and after the exposition of the films to water or acidic conditions conclusively ruled out any conductivity increase with the water content of the film. Immersion of the films in deionized water results in a marked decrease of the conductivity, also associated to a deterioration of the surface smoothness (Figure S11 and Table S2). Reduction of the conductivity is even more pronounced when films are exposed to acidic solutions (d'Ischia et al., 2013) (Figure S12 and Table S3). Notably, the films appear moderately stable under accelerated aging (Table S4), but stability is lost if the film was previously immersed in water (Table S2). In light of known literature (Bothma et al., 2008; Wünsche et al., 2015; Di Mauro et al., 2016), this behavior clearly suggests that contribution of the ionic effects in the charge transport can be considered negligible in HVAE. Moreover, the drastic effects induced by the exposition to soaking (Ito et al., 2011) water or acidic solutions witness the key role of packing of the aromatic polyindole systems in determining electrical properties of the films (Jastrzebska et al., 2002; Ito et al., 2011; Noriega et al., 2013; Liu et al., 2017).

The here observed increases in the conductivity cannot be ascribed to the formation of films akin to dense carbon black materials (Celzard et al., 2002; Jan et al., 2006), because the processes producing these materials operate high temperatures (1,000°C or more) when applied to eumelanin-like materials (Kong et al., 2012; Li et al., 2013), or anyway at temperatures above 600°C to obtain good conductivity values when applied to polypeptides rich in eumelanin precursors (phenylalanine) (Namgung et al., 2017). Instead, in this study it is observed a notably conductivity increase, from 3 to 5 orders of magnitude, even after annealing in the 200°C÷450°C range. This strongly suggests that conductivity rise has not to be ascribed to carbonization processes. Indeed, elemental analysis data (Table S5) do confirm the material does not present C/X ratios expectable for carbon black materials (Celzard et al., 2002).

Although the disordered eumelanin stuffs could suggest that the range of temperatures over which carbonization may take place is likely to be very broad, relevant literature addressing thermal evolution similar materials (Liu et al., 2007; Jin et al., 2016) (phenol and pyrrole polymers) do show no carbonization occurs below 850–900°C.

Moreover, the little observed increase in the C/X ratio is actually related to the loss of the labile CO_2_ groups (see Table S5 legend) as confirmed by the nearly constant C/N ratio.

On this basis, even the possible occurrence of a small amount of carbonization can be ruled out and, even more so, graphitization must be excluded as it requires even higher temperatures (Zajac et al., 1994; Li et al., 2013).

Measurements of electrical resistance vs. temperature were also performed (Figure S14), measuring the two-terminals devices of one type of the HVAE (600°C, 2 h, 10^−6^ mbar). The observed values of R and the trend of R vs. T reveal that not simple mechanisms are operating for the conductivity of the material: the small values of R indicate that it is a good electronic conductor (Le et al., 2017), while its trend in this range of temperatures cannot discriminate between a nature of semiconductor (decreasing R vs. T) or of conductor (increasing R vs. T), indeed a task beyond the scope of this paper.

## Conclusions

Results here reported indicate a radical modification of the actual picture of the eumelanin charge transport properties, reversing the paradigm according to which eumelanin conductivity increases with the water content of the material. Indeed, if the eumelanin films are rearranged into conductive layers, thanks to a simple thermal annealing in vacuum which succeeds in inducing a structural reorganization of their molecular constituents, the contribution of the electronic current is here demonstrated to be largely preeminent with respect to the reported ionic one (Mostert et al., 2012; Di Mauro et al., 2016; Sheliakina et al., 2018). This allows to obtain unprecedented high conductivity values, up to 318 S/cm in this work, and the mammalian pigment model, the DHI eumelanin, can be considered as an actual conductor. The conductivity values here achieved and their fine tuning, allowed by the control of the process conditions, open to possible tailoring of *ad-hoc* eumelanin-based active layers for a wide range of applications in organic electronics and bioelectronics, deserving further extensive investigations to get a conclusive picture about the conductor vs. semiconductor behavior of the eumelanin and insights about the mobility of the charge carriers.

## Data Availability

All datasets generated for this study are included in the manuscript and/or the Supplementary Files.

## Author Contributions

All authors conceived the experiments. LM and PM with contributions by AP, PT, and DA carried out the measurements. LM, AP, and PT processed and analyzed experimental data. LM fabricated all the samples. All the authors discussed the results and wrote the main manuscript. AP, PT, CG, MGM and CM contributed to refine the manuscript.

### Conflict of Interest Statement

The authors declare that the research was conducted in the absence of any commercial or financial relationships that could be construed as a potential conflict of interest.
