# Supplementary material for: Evidence of Unprecedented High Electronic Conductivity in Mammalian Pigment Based Eumelanin Thin Films After Thermal Annealing in Vacuum
Source: Front Chem. 2019 Mar 26;7:162. doi: 10.3389/fchem.2019.00162 (PMC6443883; doi:10.3389/fchem.2019.00162)
Supplement: Supplementary file 1 [file Data_Sheet_1.docx]

Electronic Supplementary Information for

Evidence of unprecedented high electronic conductivity in mammalian pigment based eumelanin thin films after thermal annealing in vacuum

Ludovico Migliaccio, Paola Manini, Davide Altamura, Cinzia Giannini, Paolo Tassini, Maria Grazia Maglione, Carla Minarini, and Alessandro Pezzella

correspondence to: alessandro.pezzella@unina.it , paolo.tassini@enea.it

**This file includes:**

Figures: S1 to S14

Tables: S1 to S5

References to conductivity of conducting polymers


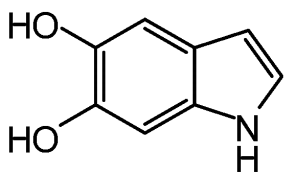


Fig. S1.

5,6-dihydroxyindole (DHI) molecule.[1]

| (a)  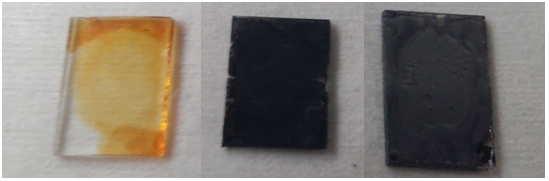 | (b)  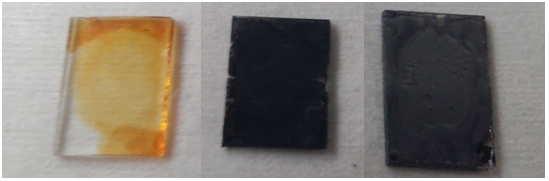 | (c)  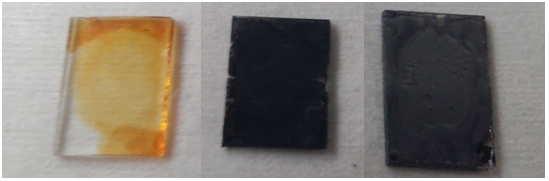 |
| --- | --- | --- |

Fig. S2.

Images of some samples of the materials prepared in this work: (a) DHI, before AISSP and no high vacuum thermal annealing; (b) DHI-eumelanin, after AISSP and before annealing; (c) High Vacuum Annealed Eumelanin (HVAE), after AISSP and after high vacuum thermal annealing (here performed at 600°C, 10^-6^ mbar, 2 h).

The DHI-eumelanin film in (b) shows the typical dark brown colour of this material.


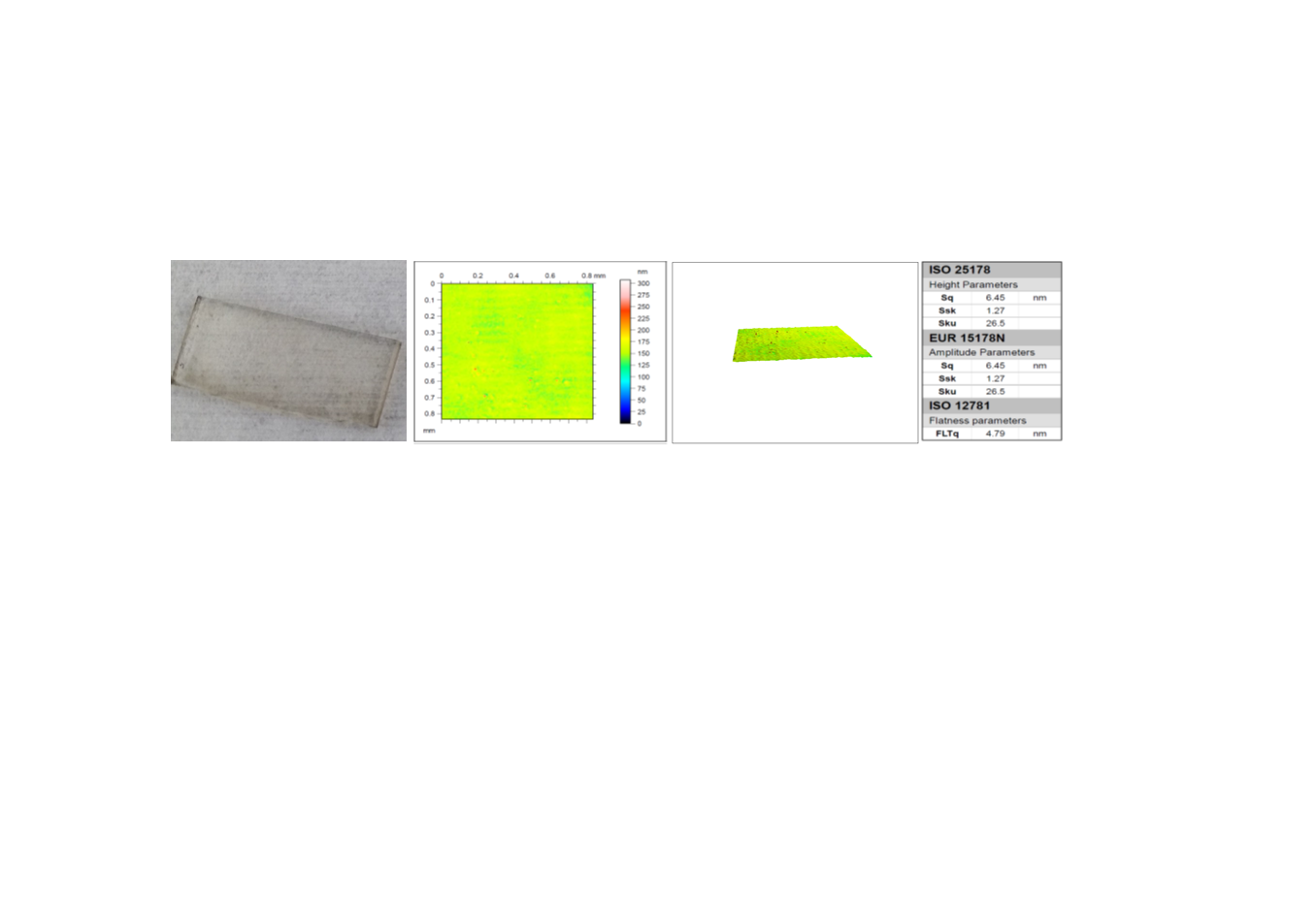


(a)


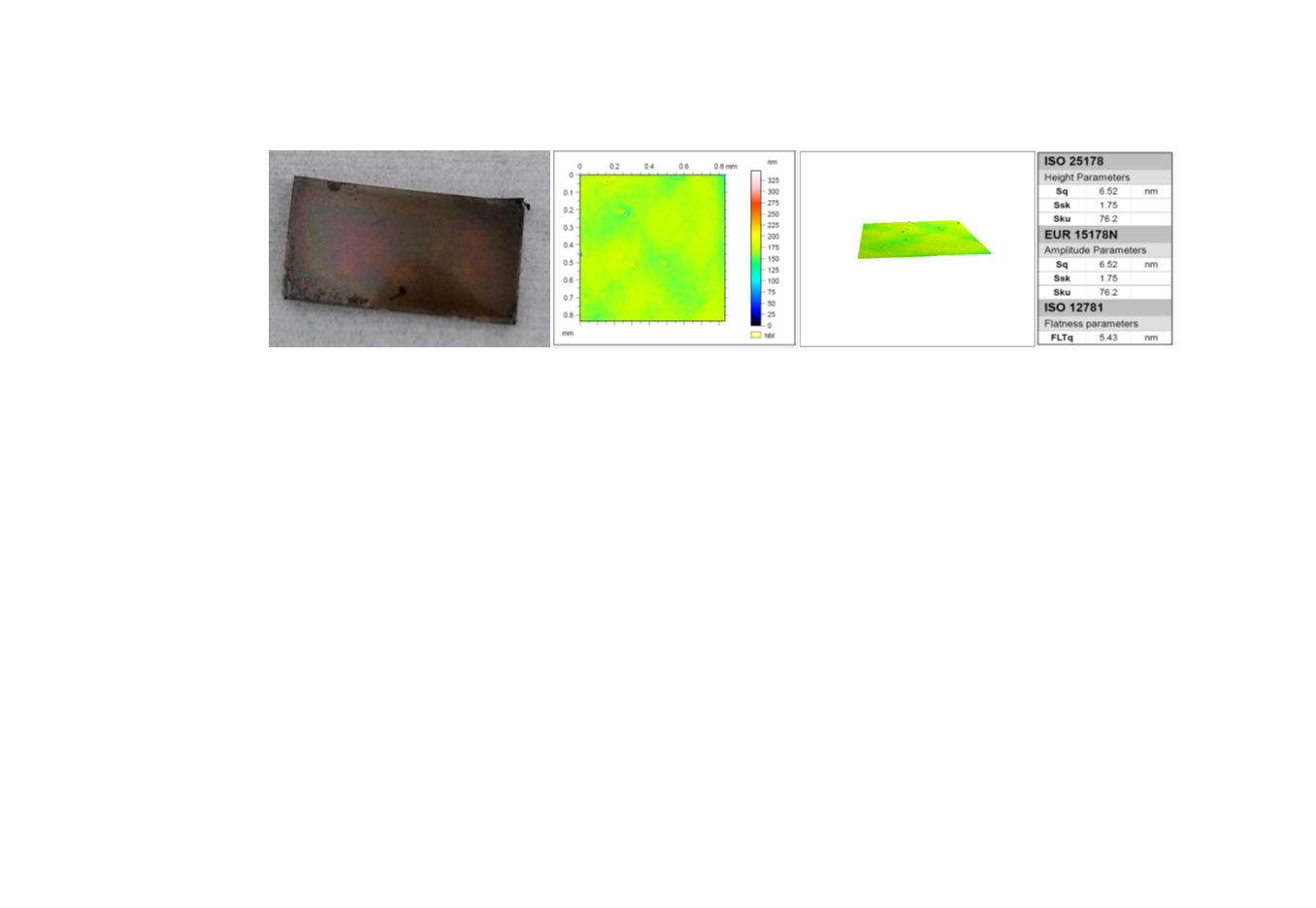


(b)


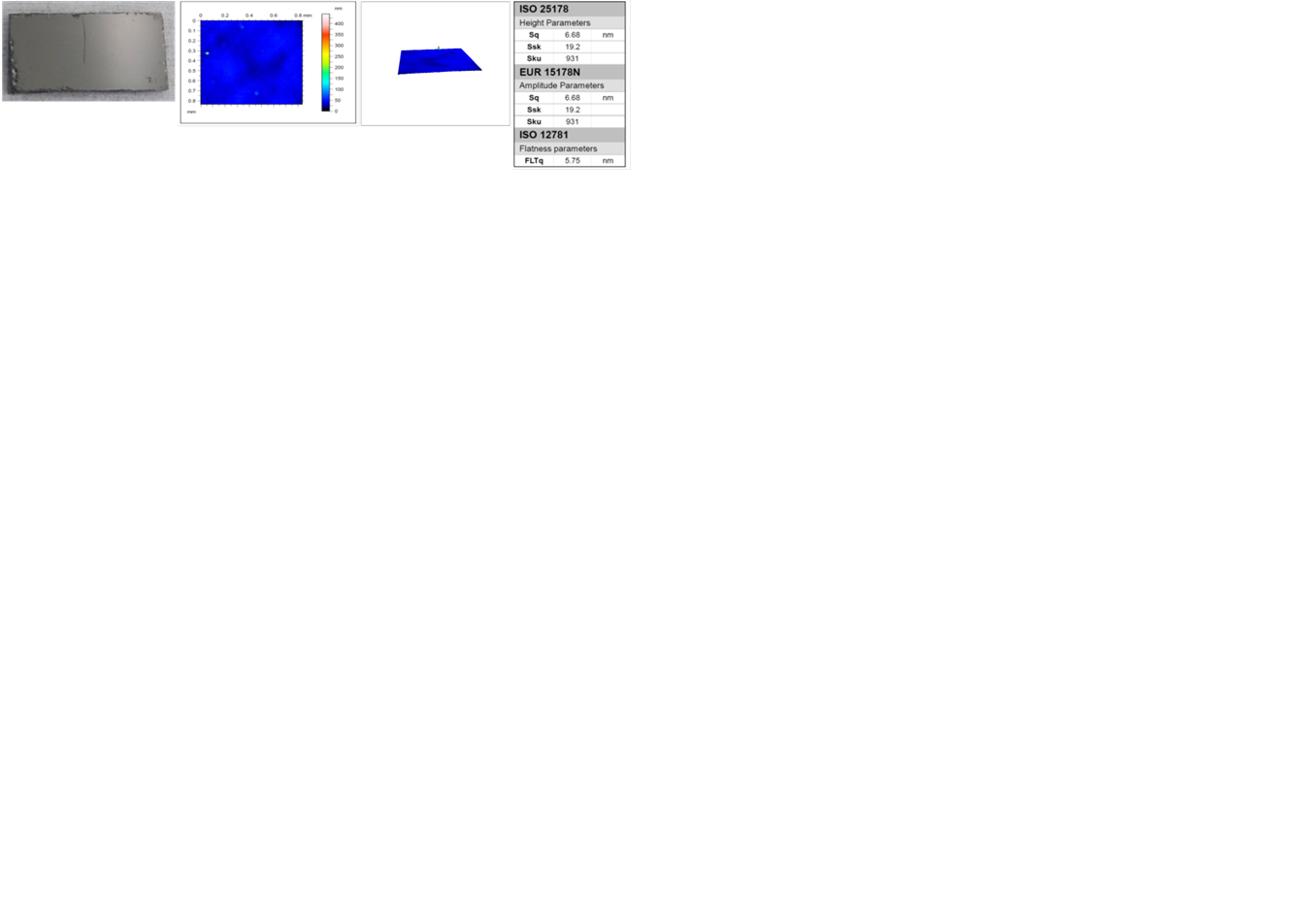

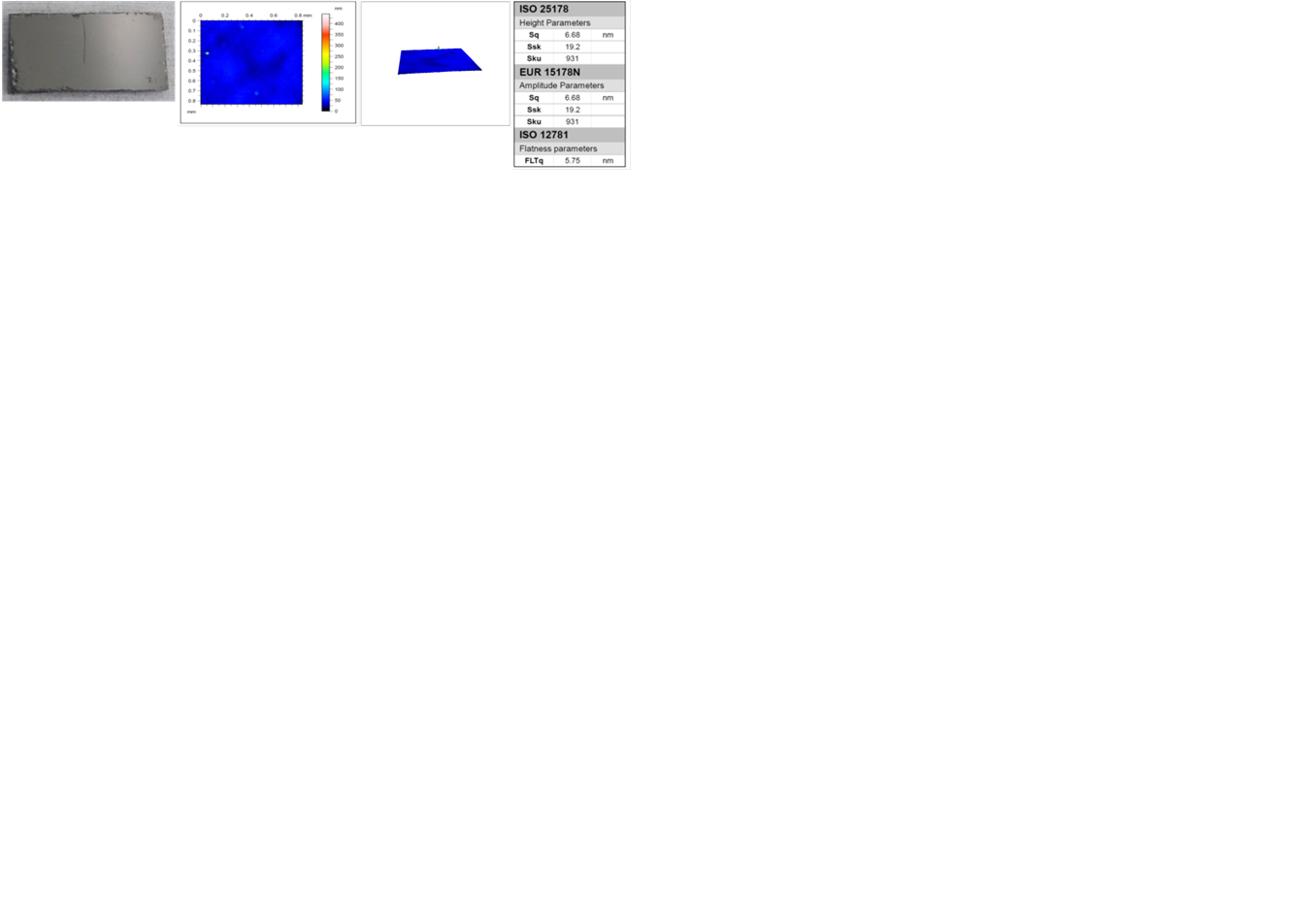


(c)

Fig. S3.

Surface profilometer characterizations of the samples of Figure S2: (a) DHI surface; (b) DHI-eumelanin surface; (c) HVAE surface. All the samples present low roughness values (*Sq* defines the roughness following the standard ISO 25178): DHI roughness = 6.45 nm; DHI-eumelanin roughness = 6.52 nm; HVAE roughness = 6.58 nm. Maximum error on these values was 6%.

(a)
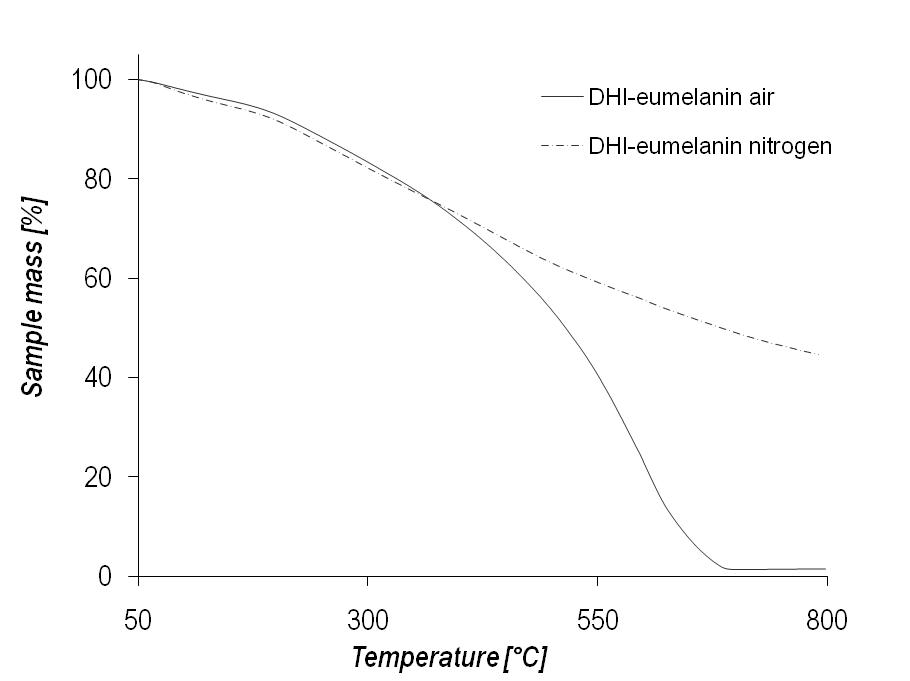


(b)
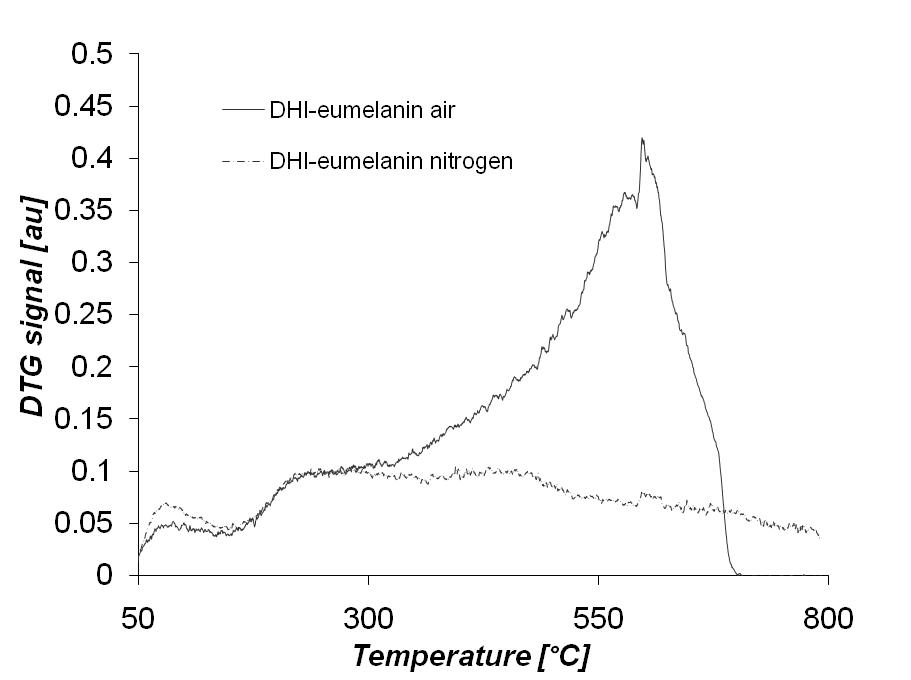


**Fig. S4.**

(a) Mass loss vs. Temperature, and (b) TGA plots for DHI-eumelanin films in air and in nitrogen. An inert environment preserves the material from a complete degradation.





**Fig. S5.**

Conductivity (large squares) and Thickness (small squares) vs. the Annealing Temperature of the HVAE films. The conductivity values are listed in Figure 4 in the main text. Here, the legend reports the thickness values. The measurements errors are inside the symbols.

(a)
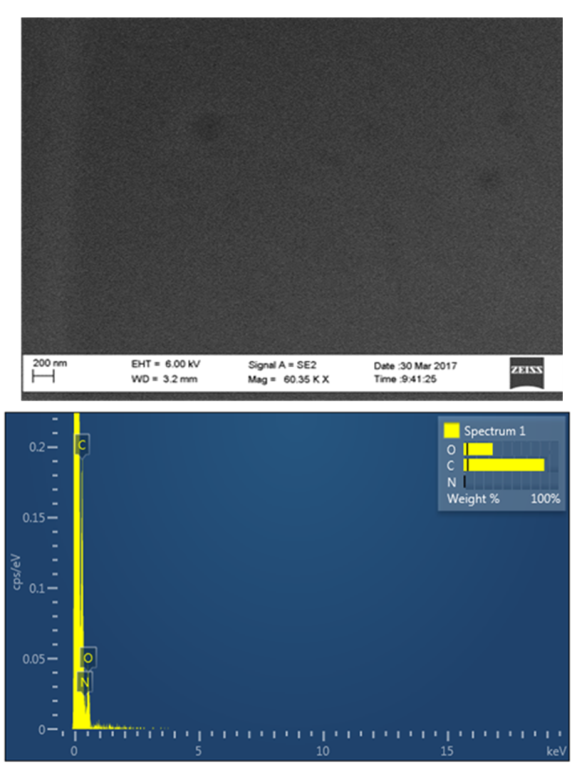




(b)
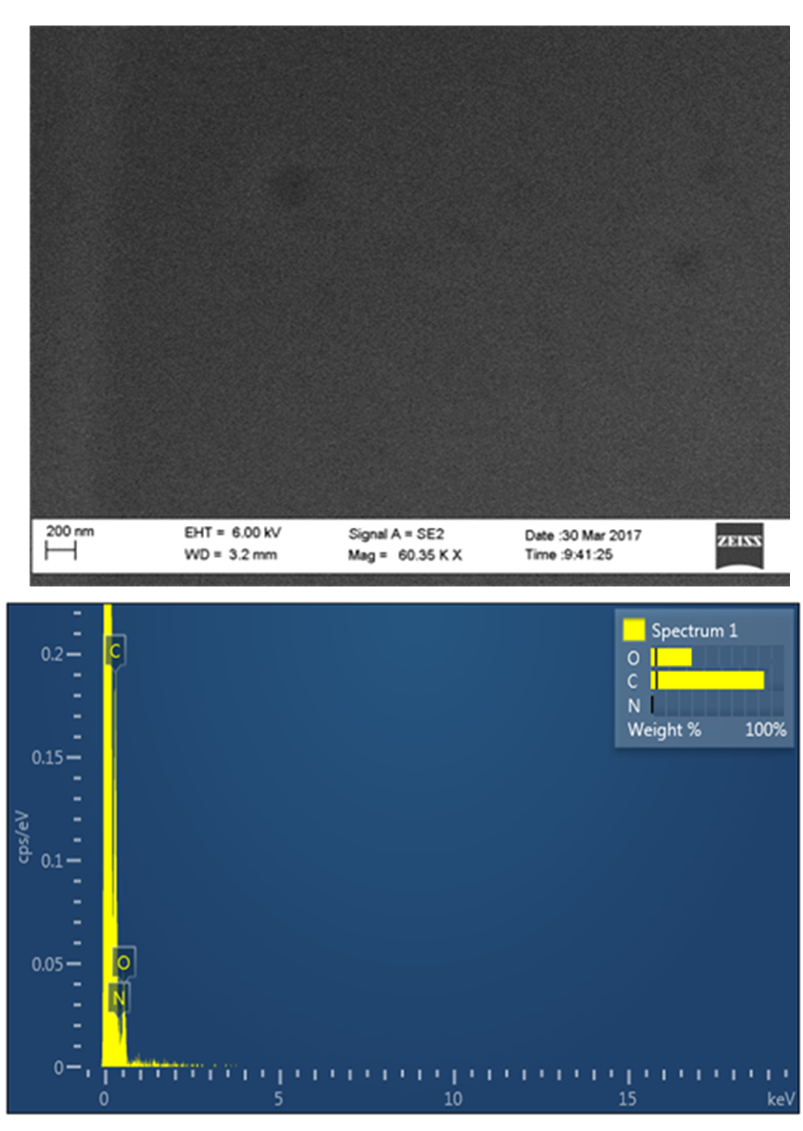


Fig. S6.

(a) SEM images (two different zones of the same sample; the visible scratch was made to measure the thickness of the film); (b) microanalysis of the HVAE treated at 600°C, 10^-6^ mbar, 2 h. All the materials’ films showed an uniform surface, while microanalysis indicated that HVAE had still a polyindolic nature.

(a)
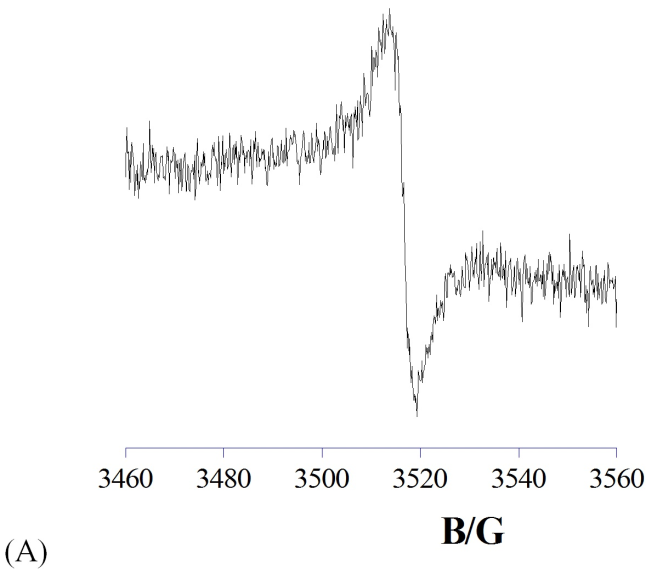


(b)
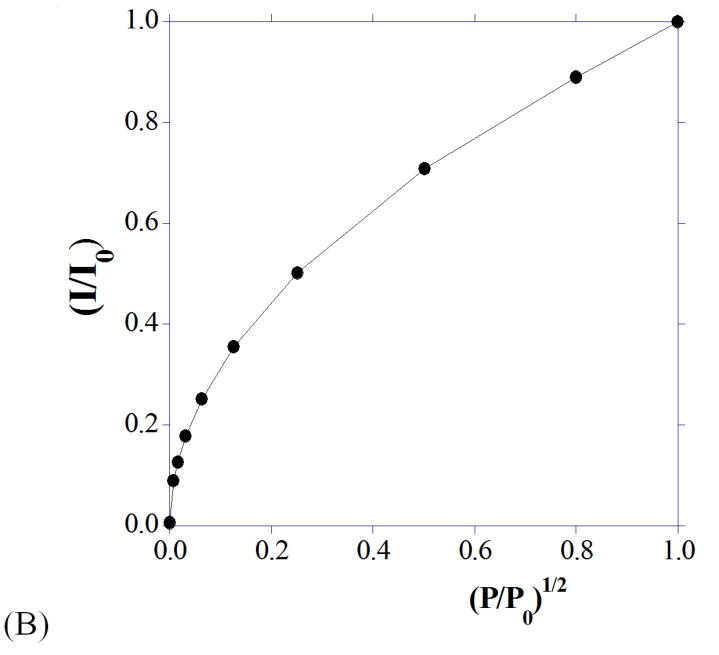


Fig. S7.

(a) EPR spectrum (x-axis: magnetic field *B* measured in gauss [G]) and (b) power saturation profile of HVAE (600°C, 10^-6^ mbar, 2 h). Data were recorded on thoroughly desiccated samples. Measurement conditions: T = 25.8°C, microbridge power = 0.6 mW.





Fig. S8.

Comparison of the Raman spectra of films of: DHI before oxidative polymerization (blue, triangles), DHI-eumelanin after AISSP (black, squares), and HVAE (600°C, 10^-6^ mbar, 2 h) (red, circles).

1150 cm^-1^


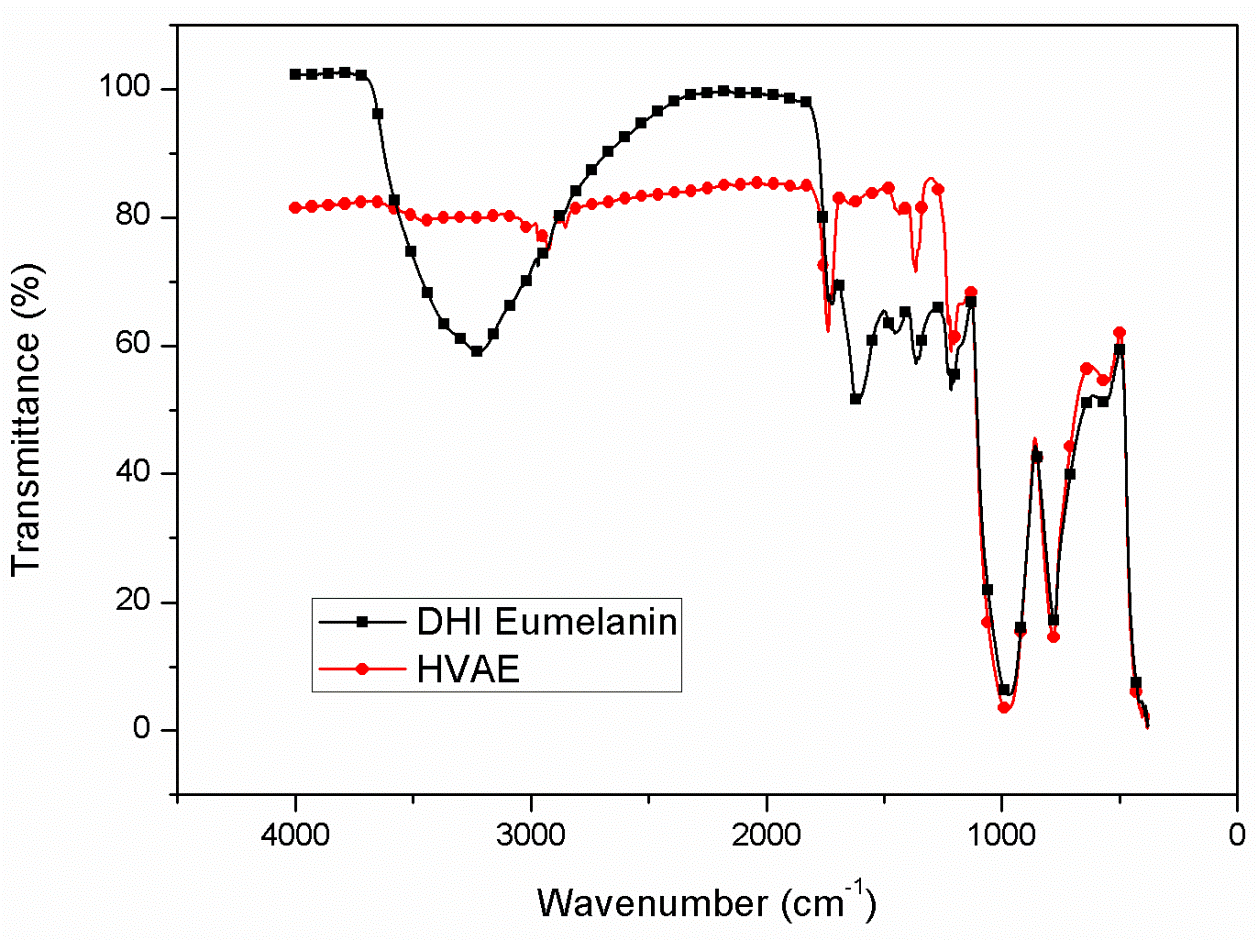


Fig. S9.

FTIR spectra of DHI-eumelanin (black, squares) and of HVAE (red, circles). Arrows denote signals associated to water (3200 cm^-1^), hydrogen bonds and carboxyl groups (1620 cm^-1^). The region with wavenumber smaller than 1150 cm^-1^ (at the right of the dotted line) is dominated by the quartz substrate signal.


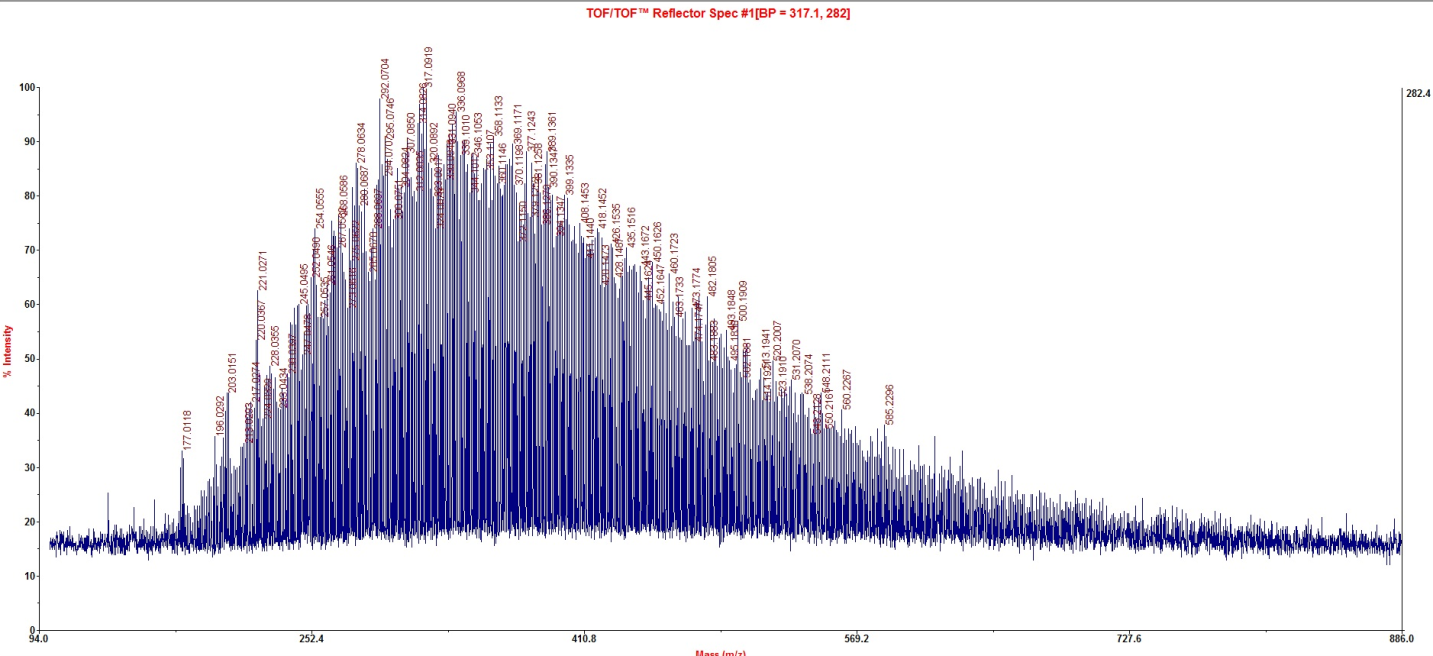


(A)


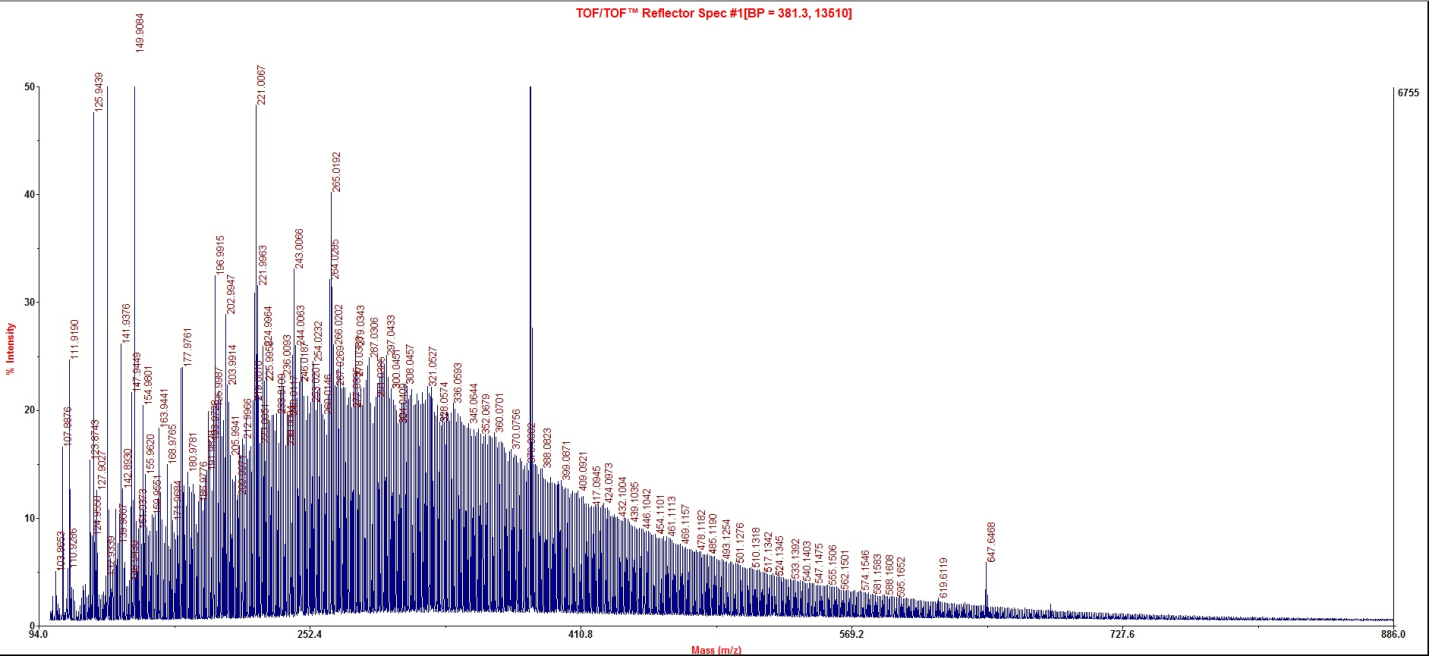


(B)

Fig. S10 (A), (B).

MALDI-MS spectra of (A) DHI-eumelanin, and (B) HVAE after high vacuum annealing. The recurring profile of masses of general formula *DHI oligomer + rnO_2_ - nCO_2_* are recognizable in both the spectra for low molecular weights [2].


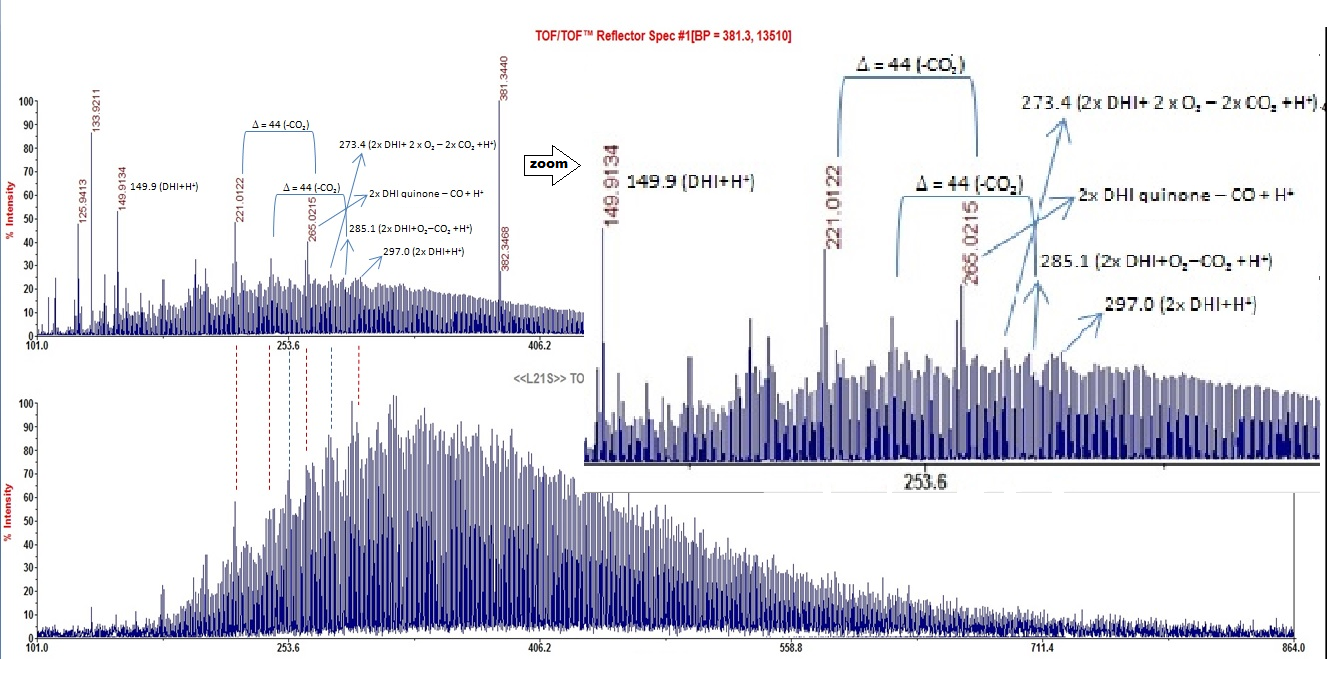


(C)

Fig. S10 (C).

Some relevant signals are highlighted in panel (C)

(a)


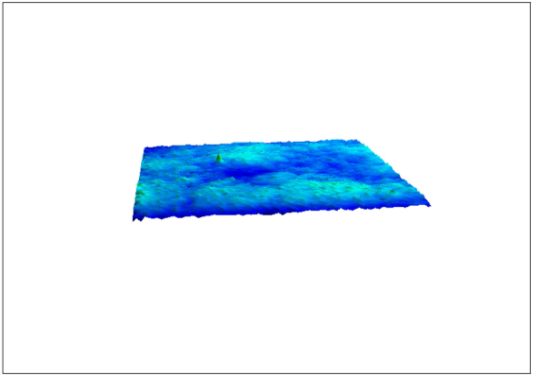


(b)


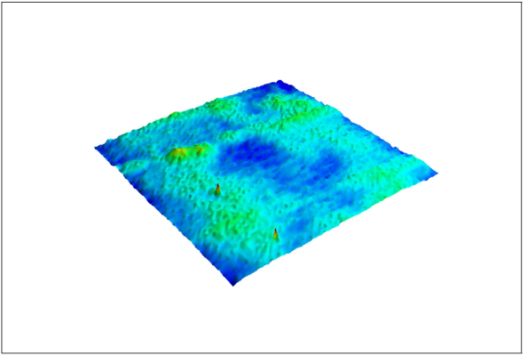


Fig. S11.

Optical profilometer surface analysis of HVAE: (a) before and (b) after 2 h immersion in deionized water (18 MΩ*cm) at room temperature. All the roughness values increase after the immersion.

(a)


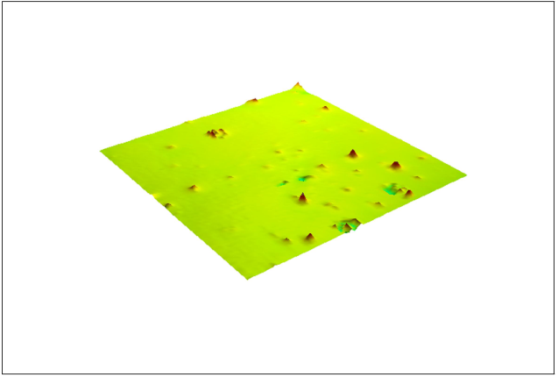


(b)


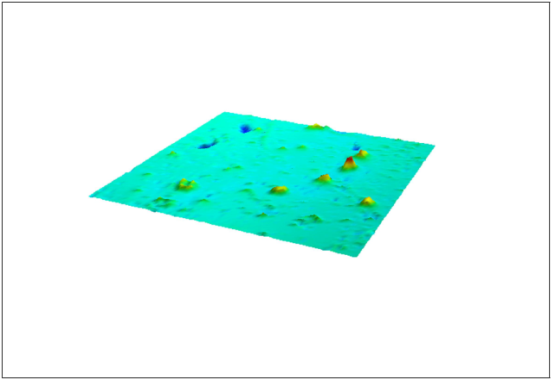


Fig. S12.

Optical profilometer surface analysis of HVAE: (a) before and (b) after immersion in a sulfuric acid solution (96 w/w%) for 2 hours at room temperature.


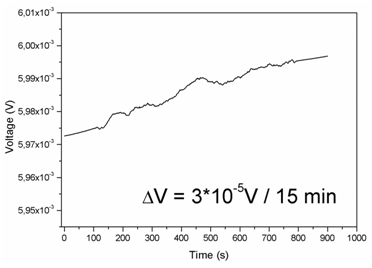


Fig. S13.

Voltage vs. Time in HVAE layers (annealing conditions: 600°C, 2 h, 10^-6^ mbar). This plot shows the trend of the voltage required to sustain a fixed current (100 μA) in a HVAE thin film (thickness = 109 nm), using two probes with 1 mm spacing, measured at room temperature in air.


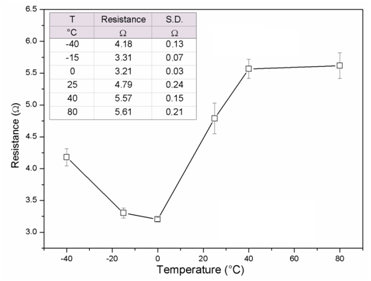


Fig. S14.

Resistance vs. Temperature in HVAE layers (annealing conditions: 600°C, 2 h, 10^-6^ mbar), showing both the small values of the films’ electrical resistance and the not univocal nature of conductor or of semiconductor of the material in this range of temperatures.

Table S1.

Materials preparation recipes and resulting thicknesses of the films.

| Material | Preparation | Resulting  Thickness  (nm) |
| --- | --- | --- |
| DHI | DHI 50 mg/mL 1:1 v/v MeOH:AcOEt  Spin recipe: 2000 rpm/s & 3500 rpm for 30 s | 230 +/- 10 |
| DHI-eumelanin | AISSP: DHI films inside a sealed chamber containing air saturated of ammonia, from 5% NH_3_ in H_2_O, for 12 h at 25°C | 260 +/- 6 |
| HVAE | High vacuum thermal annealing of the DHI-eumelanin films at 600°C and 10^-6^ mbar for 2 h | 110 +/- 2 |

Table S2.

Thickness, Sheet Resistance and Normalized Conductivity values of one type of the HVAE samples (600°C, 2 hours annealing), measured before and after water immersion and after different treatment conditions. Errors are in the limit of 2% for each datum.

| HVAE  (600°C, 2 h) | Before 2 h  water  immersion | After 2 h  DI water  immersion | After three  weeks at  standard  humidity  and  temperature  conditions | After  climatic  chamber  treatment:  *RH* = 85%  *T* = 65°C  time = 90 h | After  annealing  on  hot plate:  in air  *T* = 115°C  time = 5 h |
| --- | --- | --- | --- | --- | --- |
| Thickness  (nm) | 96.8 | 96.8 | 105.4 | 109.2 | 103.7 |
| Sheet  resistance  (Ω/□) | 415 | 540 | 1670 | 1850 | 1441 |
| Normalized  Conductivity  (%) | 100 | 76.4 | 22.80 | 19.80 | 26.80 |

Table S3.

Thickness, Sheet Resistance and Normalized Conductivity values of one type of the HVAE samples (600°C, 2 hours annealing), measured before and after sulfuric acid treatment. Errors are in the limit of 2% for each datum.

| HVAE  (600°C, 2 h) | Before H_2_SO_4_ treatment for 2 h | After H_2_SO_4_ (96 w/w% in DI water) treatment for 2 h |
| --- | --- | --- |
| Thickness  (nm) | 120 | 161 |
| Sheet resistance  (Ω/□) | 342 | 404 |
| Normalized  Conductivity  (%) | 100 | 63.1 |

Table S4.

Thickness, Sheet Resistance and Normalized Conductivity values of one of the HVAE samples (600°C, 6 hours annealing), measured immediately after the preparation process and after different treatments or storage conditions. Errors are in the limit of 2% for each datum.

| HVAE  (600°C, 6 h) | After  preparation  process | After three weeks  at standard  humidity  and temperature  conditions | After climatic  chamber  treatment:  *RH* = 85%  *T* = 65°C  time = 90 h | After  annealing  on hot plate:  in air  *T* = 115°C  time = 5 h |
| --- | --- | --- | --- | --- |
| Thickness  (nm) | 95.5 | 115.7 | 121.2 | 118.1 |
| Sheet resistance  (Ω/□) | 340 | 440 | 528 | 527 |
| Normalized  Conductivity  (%) | 100 | 62.07 | 49.67 | 51.93 |

Table S5.

Elemental analysis, estimated by a Perkin–Elmer 2400 CHNSO elemental analyzer, of the various samples prepared . Elemental composition wt% (average of duplicate measures) and element ratios of: DHI, DHI-eumelanin as prepared, and HVAE samples. Errors are in the limit of 2% for each datum. It is worth of note how the relative oxygen content increases after the DHI oxidative polymerization and decreases with thermal treatment. The relative ratios of the elements may be interpreted as a consequence of the loss of water and CO_2_. The values obtained after the more extensive treatment does not match the ones of ordinary carbon black materials. [3]

| Sample | %C | %H | %N | %O | C/O | C/N | N/O | (C+N)/O |
| --- | --- | --- | --- | --- | --- | --- | --- | --- |
| DHI | 63.81 | 4.01 | 8.14 | 24.02 | 3.54 | 9.14 | 0.38 | 2.28 |
| Eumelanin | 50.53 | 2.28 | 5.87 | 41.3 | 1.63 | 10.02 | 0.16 | 1.04 |
| HVAE  (231°C, 12 h) | 59.65 | 2.01 | 7.12 | 31.22 | 2.54 | 9.77 | 0.26 | 1.63 |
| HVAE  (600°C, 6 h) | 57.34 | 2.98 | 9.13 | 30.55 | 2.50 | 7.32 | 0.34 | 1.67 |

[1] R. Edge, M. D'Ischia, E. J. Land, A. Napolitano, S. Navaratnam, L. Panzella, A. Pezzella, C. A. Ramsden and P. A. Riley, *Pigment Cell Research*, 2006, **19**, 443-450.

[2] A. Napolitano, A. Pezzella, G. Prota, R. Seraglia and P. Traldi, *Rapid Commun. Mass Spectrom.*, 1996, **10**, 468-472.

[3] B. E. Barton, E. J. Hukkanen, G. F. Billovits, D. Schlader, M. J. Behr, D. Yancey, M. Rickard, X. Qiu, D. M. Mowery, L. Brehm, B. Haskins, W. Wang, M. S. Spalding and C. Derstine, *Carbon*, 2018, **130**, 288-294.

References to conductivity of conducting polymers [^1-3^](#_ENREF_1)

1. T. H. Le, Y. Kim and H. Yoon, *Polymers*, 2017, **9**.

2. M. H. Naveen, N. G. Gurudatt and Y. B. Shim, *Applied Materials Today*, 2017, **9**, 419-433.

3. T. Nezakati, A. Seifalian, A. Tan and A. M. Seifalian, *Chemical Reviews*, 2018, **118**, 6766-6843.
